# The use of models and modelling in design projects in three different technology classrooms

**DOI:** 10.1007/s10798-022-09730-9

**Published:** 2022-02-02

**Authors:** Björn Citrohn, Karin Stolpe, Maria Svensson

**Affiliations:** 1 Department of Physics and Electrical Engineering, Linnaeus University, Växjö, Sweden; 2 Department of Behavioural Sciences and Learning (IBL), Linköping University, Linköping, Sweden; 3 Department of Pedagogical, Curricular and Professional Studies, University of Gothenburg, Gothenburg, Sweden

**Keywords:** Models, Technology education, Activities, Design project, Dimensions of models

## Abstract

In this study, we aim to investigate activities using models in a design project in three technology classrooms. Activities that use models are important for students’ development of knowledge and skills connected to the design process. Nevertheless, few empirical studies have thus far examined how models and modelling are used in a classroom environment when students and teachers are involved in a design project. In order to meet our aim, we video-recorded eight lessons from three different technology classrooms (students aged 13–15), where the students were involved in different problem-solving activities using models and modelling. The three projects had different specifications, and the students’ degrees of freedom thereby varied. The video recordings were analysed using a qualitative content analysis. The analysis resulted in seven activities being identified where the teachers and students talked about models and modelling in order to solve the problem. The results also revealed three different dimensions of models: material, structure and function. These dimensions are present in almost all activities that use models. In a project with a high degree of freedom, all three dimensions of models are present. On the contrary, in a project with a lower freedom, only one of the dimensions is present, resulting in a lower degree of complexity for the students. The study emphasizes that the presumptions and openness of a design project in technology education can provide different possibilities for students learning in relation to models and modelling.

## Introduction

Technology as an activity relates to the development of solutions to practical problems (Rodney et al., 2001), often described as *designing*, or the *design process*: a process of experimenting, trial and reflection, exploring, and decision making (Dooren, et al., 2019). Governments in different countries often promote the “innovative society”, in which design is a crucial element (Anthony et al., 2012). Students need to develop their skills, knowledge, methods and technologies so that they will be able to evolve an innovative society in the future, but also for the advancement of industry (Anthony et al., 2012). Modelling, a process used in the design process, is representational, explanatory and predictive (Rossouw et al., 2010). France et al. (2011) emphasize that a key aspect of developing students’ technological literacy is to enhance their conceptual understanding of modelling.

When carrying out activities like design and modelling, knowledge is needed and at the same time design activities are knowledge-generating (Rauscher, 2010). In an analysis of Swedish compulsory school students’ descriptions of technology, Svenningsson (2019) emphasizes the importance of testing (modelling) and discussing different solutions when designing in order to increase students’ technological knowledge.

The present study is situated in three Swedish technology classrooms, where the students work on design projects involving modelling. The teachers and students use models in different ways throughout the process. Hence, this study seeks to develop knowledge about which aspects of technology become available to students within design projects.

The combination of the two processes of *designing* and *modelling* is used in many countries’ technology curricula. One example is the New Zealand curriculum for technology which describes technology as intervention by design and that the subject contains various forms of technological modelling (Ministry of Education, 2021). The Swedish curriculum for technology in compulsory school contains a design process called *technology development work*, which includes formulating problems, analysing and constructing ideas, and using sketches, drawings and physical models. Being able to communicate through sketches, drawings and models, both physical and digital, as well as interpreting them, is connected to visual thinking skills. These skills are seen as an important part of problem-solving (Skolverket, 2021a). The design process as such is a model of how to handle and solve technological problems however, in the present study the design process as a model is not the research subject in focus, instead we want to study if and how models are used as part of the process.

However, the role of modelling in designing has not undergone a proper analysis given that modelling is the “language” of designing (Roberts et al., 1992). A study by Welch (1998) investigated how untutored year 7 students use three-dimensional modelling when designing a solution to a technological problem, and emphasized the role of teachers in guiding the students throughout the project. Welch concluded that it is important to explore and evaluate solutions early in the design process by using three-dimensional models.

An interview study of teachers’ perceptions of model functions in technology education showed that teachers focus on using model functions as representations and do not use models when solving problems or evaluating or modifying models (Citrohn & Svensson, 2020). Based on the results from the interviews with teachers, Citrohn & Svensson, suggest further examination of the use of models in classroom activities in the technological design process.

Prior research indicates that the use of models and modelling activities are important for students’ development of technological knowledge and skills, especially in connection with the design process. However, there are few empirical studies examining which activities using models are present and which role these activities play during a design project in a technology classroom. The present study aims to contribute to the knowledge about activities that use models within the design process when working with a design project in a technology classroom. To meet this aim, the study will answer the following overall research questions:

• Which activities using models are explicated in relation to modelling in the design process?

• What role do activities using models play in a design project?

## Theoretical background

Since this study aims to investigate the *activities using models* in education when teachers and students are involved in a design project, we will apply a framework involving models and modelling as a foundation. In the following sections, we begin from a wide perspective, *the philosophy of technology*, move on to the *nature of models and modelling*, and end with *earlier research about models and modelling in technology education*.

### Philosophy of technology in relation to design and models

The technology philosopher Mitcham retains a view of technology from both engineering and humanities philosophy, which means that he puts the human being at the centre of what technology is. He defines four manifestations of technology: technology as *objects*, *knowledge*, *activities* and *volition* (Mitcham, 1994). When examining the Swedish curriculum for technology (Skolverket, 2021a) with focus on the design process, it appears that there are links to Mitcham’s aspects of technology. The aspects of *objects* are understood in a broad way as individual objects and as systems of connected objects. The most common way of describing technology is as an object (Svenningsson, 2019). In relation to the design process in the Swedish curriculum, objects can be connected to *constructions to describe the students’ own technical solutions*. The activity aspect is, in the simplest terms, described as the making and use of technological objects. In relation to the Swedish curriculum, it is often connected to building and constructing. Svenningsson (2019) shows that Swedish students tend to describe technology as “building things” and “inventing”, which has strong connections to the design process.

An article by Ankiewicz et al. (2006), departing from Mitcham´s four manifestations, was paying special attention to *knowledge* and *activity*. When investigating the latter, technology as activity, the authors define technology as both *complex thinking* and *practical activities* both included in the design process.

One reason why the subject of technology in Sweden is connected to building or creating (Skolinspektionen, 2014) might be the history of the subject, with its focus on developing handcraft skills (De Vries & Tamir, 1997). *Knowledge* is, according to Mitcham, within humans and is needed for making and using objects, and is thus important for the design process in two ways. The humans involved in the design process must have knowledge about how to use the artefacts and tools needed in the process. Moreover, they need to have knowledge of making or developing the artefact, which is the goal of the design process. One type of knowledge is *sensorimotor skills*, which is used when making things. This type of skill could be connected to *know-how* (Ropohl, 1997), which implies cognitive resources that the human consciousness is not usually explicitly aware of and that can only be gained through practice. Sensorimotor skills, which are acquired through trial and error and intuition, are often associated with craftsmen or carpenters (Mitcham, 1994). Another example of *knowing how* is the type of knowledge engineers express in their sketches and drawings. Regarding *knowledge* Ankiewicz et al. (2006) define knowledge as *conceptual knowledge* and *procedural knowledge.* The latter includes design, modelling, problem solving which all are connected to the design process. The last aspect of Mitcham’s definition of technology, *volition*, is described as the human will to survive, control and make our life more efficient. It is present in the Swedish curriculum defining the aims and purpose of the subject of technology. When students work practically with their own technical ideas and solutions and use technology’s forms of expression, this contributes to their development of abilities to tackle technical challenges in a conscious and innovative way. Technology education aims to engage the students through challenges when designing models and products in order to find new solutions.

Svenningsson (2019) argues that it is important to include all four of Mitcham’s aspects of technology in education. In doing so, students have the opportunity to develop their technological knowledge. The design process as described in the Swedish curriculum also allows for the development of the four aspects. Next, we will examine the nature of models and modelling, and will draw a distinction between models and prototypes and their use in technology.

### The nature of models and modelling

In technology, models are often used for visual support when solving problems, to predict actions or events, to make decisions, and to communicate (Welch, 1998). The model is tested, submitted to changes, and possibly tested again. This process can be described as modelling. Welch (1998) proposes that the term model, as a noun, is more commonly used in its active form – “modelling” – in the context of technology education. Modelling makes ideas more accessible to oneself and others, and it also facilitates testing and evaluation (de Vries, 2013; Norström, 2013; Welch, 1998).

**Fig. 1:**
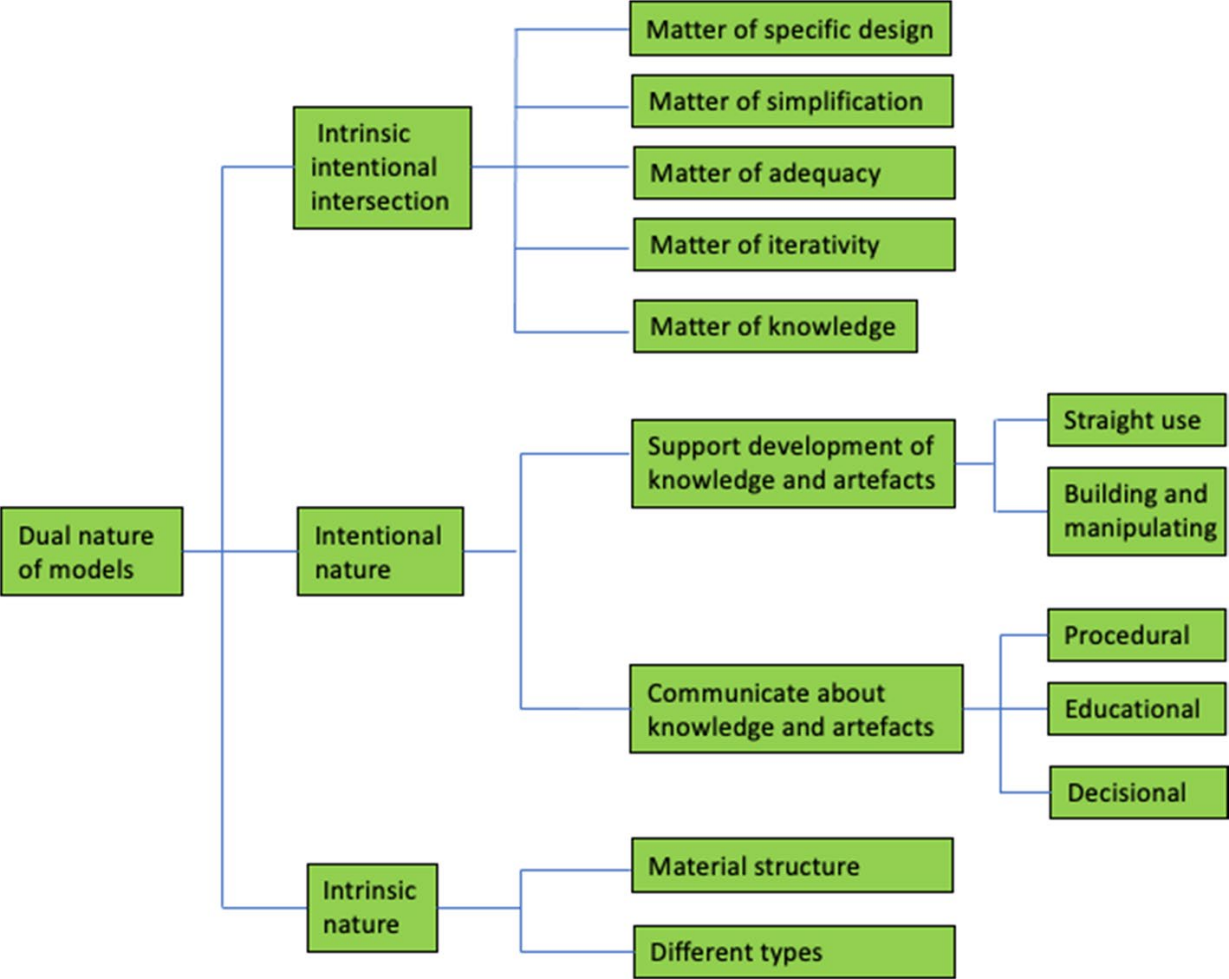
The framework of the dual nature of models (based on Nia & de Vries, 2017)

An interesting philosophical description of models is that of *models as artefacts of dual nature*, a framework that identifies models as having one *intrinsic* nature and one *intentional* nature (Nia & de Vries, 2017). The intrinsic nature is about the material structure and different types of models, while the intentional nature is about the representational task of models. This framework will be used to analyse the results in our study. The intentional nature of models is of particular importance, since it can be connected to *activities* carried out by the designer (the student) when developing the model. The *intentional nature* of models (Fig. 1) consists of two intentions: *supporting the development of knowledge and artefacts* and *communicating about knowledge and artefacts* (Nia & de Vries, 2017). The first intention, *supporting the development of knowledge and artefacts*, is connected to acquiring knowledge about designing and building, or how to optimize certain artefacts. It consists of two ways of using models: *straight use* and *building and manipulating.* The second intention, *communicating about knowledge and artefacts*, is another important function for using models when communicating with teams, students, decision makers or customers, for example. The *relationship between the intrinsic and intentional nature of models* is also interesting for our analysis of the activities using models. Starting from the user’s view and the designer’s view of models, Nia and de Vries (2017) emphasize five different matters: *the matter of specific design of models*, *the matter of simplification in models*, *the matter of iterativity in modelling*, *the matter of adequacy of models* and finally *the matter of knowledge behind models*.

### Earlier research about models and modelling in technology education

In technology education, modelling is important to students for a wide range of purposes: Visualizing the whole or component parts and appearance of the final product, testing the performance of solutions like mechanisms and circuits, testing ergonomics, improving the form of the product, communicating ideas and informing others and evaluating ideas of others (Welch, 1998).

In a school context, students often build simplified models of the intended final product. The models are typically made from everyday materials like cardboard, wooden sticks, plastic film and paper (see e.g. Yrjönsuuri et al., 2019) classifying them as Soft Models or Hard Models (Isa & Liem, 2014). Furthermore, the models typically have only a few of the final product’s functions because building them would be too complicated or too expensive. *Soft Models* are typically used for rough modelling to assess the overall size, proportion, and shape and are refined by hand. *Hard models* are technically non functional replicas of the final product and have some working features (Isa & Liem, 2014). Both can be used for proof-of-concept in early stages of development.

Swedish technology teachers mainly connect model functions to the design process in order to explain and facilitate understanding technological solutions (Citrohn & Svensson, 2020). In the design process, the models are used in a prescriptive way in order to develop new technological solutions. In the context of *explain and facilitate understanding of technological solutions*, the use of models is closely related to a scientific descriptive way of using models. One conclusion from the study was to address the importance of using models in the technological design process, to *emphasize the prescriptive way of using models in technology* (Citrohn & Svensson, 2020).

In order to define our perception of models in a school perspective, we need to establish the difference between the two concepts: models and prototypes. The two concepts are often confused and combined in the school context. Based on our interest in activities, we examine two activities: modelling and prototyping. The two activities are considered quite different activities in professional industrial design. Prototyping can be defined as a method that uses physical prototypes to study how a new product will be used and what it will look like (Hallgrimsson, 2012) or for problem solving, a kind of culture and language (Kelley, 2001). Modelling can be defined as a logical step in the thinking process for a design idea (Terstiege, 2012). When someone starts to use materials, they are able to refine their ideas better. Within materialization, prototypes will mostly be used to test and measure the final design proposal with respect to design specifications and to make sure that it functions, both technically and from a user perspective (Isa & Liem, 2014).

Prototypes are not mentioned in the Swedish curriculum, but modelling is the final step in the design process; *The phases “proposal for solutions”, “construction” and “testing” in technology development work in the school are, in a similar way to in engineering methods, a synthesis that shows the students’ understanding in the form of sketches, drawings, descriptions and physical or digital models. Once again, it is emphasized that the students’ work probably does not lead to production, but through their documentation they show their understanding of the technology that has been treated on the basis of the assignment.* (Skolverket, 2021b p. 5).

## Method of the study

This study follows a qualitative, generic inductive approach (Liu, 2016) and is explorative in its nature. The data are based on video observations. In line with the generic inductive approach (Liu, 2016), the sampling is purposive. Technology classrooms in different Swedish secondary schools, grades 7–9 (students aged 13–15) were selected for the observations. All three teachers were fully licensed, experienced technology teachers. They were selected since they were teaching a design project at the time of the data collection. However, all three teachers had chosen different content for their projects. Technology education in Sweden includes solving technical problems using an approach with the following steps: identification of needs, investigation, proposed solutions, construction and evaluation (Skolverket, 2021b). Later, the contexts and design projects of the different cases will be described in more detail.

Data were collected between April and December 2020. During this time period, the Covid-19 pandemic affected both the schools and the data collection. Due to sick leave among the teachers, only two out of three lessons were recorded in the Pedometer case. In the autumn, when the Greenhouse case and the Bridge case were recorded, the schools were closed to outside visitors. The teachers were therefore lent video-recording equipment consisting of a camera in the form of an iPad, mounted on a robot that rotates to follow the teacher. The teacher wore a microphone and a remote control which the robot detected. When the teacher moved within the classroom, the robot rotated to capture the teacher on video. The robot was placed in one of the corners at the back of the classroom. All three lessons were video-recorded for the Greenhouse case and the Bridge case. In total, about 7 h of video recordings were collected.

Below, the three different classrooms are described in greater detail as three different cases. In Sweden, teachers are free to choose the content of lessons, as long as the core content and knowledge specifications are fulfilled. The three different teachers all work with design projects. However, they do so in different grades, and with different content. Hence, the three classrooms represent a variation of design projects in Swedish technology education.

### The pedometer case

In the first case, the students were asked to design a pedometer controlled by a microcontroller. The project was conducted as a collaboration between the two subjects of technology and arts. The technology teacher, together with the arts teacher, led the project in grade 8 during the 4–5 weeks for which the project lasted. The class had two 60-min lessons per week. The students had prior knowledge about control and regulation from grade 7, and several students had experience of programming microcontrollers. Before starting the project, the students worked theoretically with design and product development, following material from the Association of Swedish Engineering Industries (Teknikföretagen, 2021). The material aimed to give the students the opportunity to work as engineers and entrepreneurs.

The project was introduced by the teacher during the first video-recorded lesson. The students were asked to design a pedometer that could be used to encourage youngsters to exercise. The teacher had made up a project brief that the Swedish National Board of Health and Welfare had formulated the mission. Moreover, representatives from the organization would come to visit an exhibition of the pedometers at the end of the project. During the exhibition, the students should also market their products. The aim of this twist was to make it more authentic for the students.

The students had access to everyday materials like cardboard, wooden sticks, glue, textile cord, plastic and small metal pieces. At the beginning of the project, the teacher showed and talked about the materials the school could provide. She also told the students that they could ask the craft teacher for materials or bring things from home. The students had access to different tools such as scissors, glue guns, pliers and knives. If other tools were needed, they could borrow them from the crafts classroom.

The pedometer also contained a microcontroller, in this case a BBC micro:bit. The students programmed a micro:bit to count steps when it was shaken. All students had their own personal computer that was used for programming. They also had more than one micro:bit per student, allowing them to keep the same micro:bit throughout the whole project. Each student designed and programmed their own pedometer. Due to the pandemic, the students were not allowed to work in pairs or groups. Even so, they helped each other out, especially with the programming part.

During the project, each lesson started with a short summary of the aims of the project. The teacher also kept reminding the students to write about their process and progress in their logbooks, both on paper and digitally. They could also draw sketches and drawings, and write notes about their progress.

**Fig. 2:**
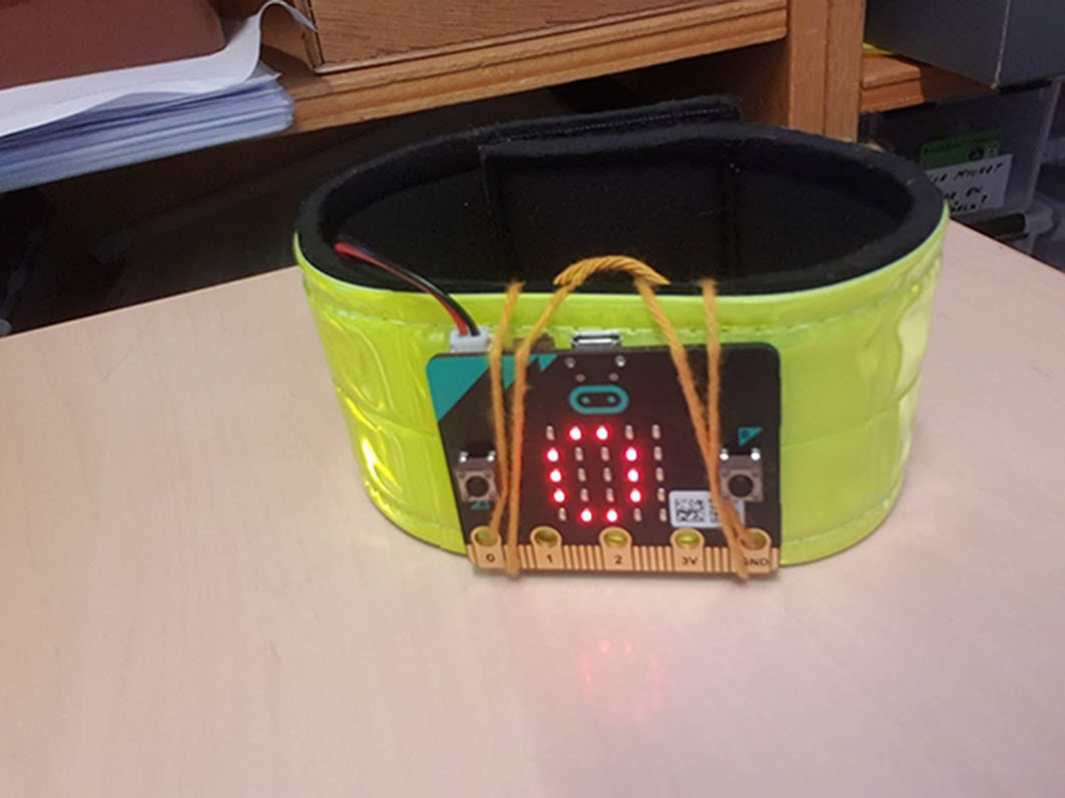
Prototype of a pedometer with a built-in micro:bit

When the project ended, each student promoted the functions and features of their pedometer model to a jury (Fig. 2). The teacher had extensive teaching experience and was one of two teachers working with technology at the school. In addition to technology, she also taught maths and science, since the class was in grade 7. This meant that she had good knowledge about the students’ prior experience and knowledge in the area.

### The greenhouse case

In the second case, the students were asked to design a miniature greenhouse with functions controlled by a microcontroller. The project lasted for five weeks, with two 60-min lessons per week. The students were in grade 9 (15 years old), and had some prior knowledge of programming. Their programming skills were also refreshed before the project began. The project was introduced by the teacher. The students could choose which functions they wanted to implement, such as automatic window opening if the temperature exceeded a certain value, or a light bulb that came on if it was too dark. At the end of the project, the students showed their greenhouse and explained and demonstrated the functions in front of the class. The students worked in groups of four.

**Fig. 3:**
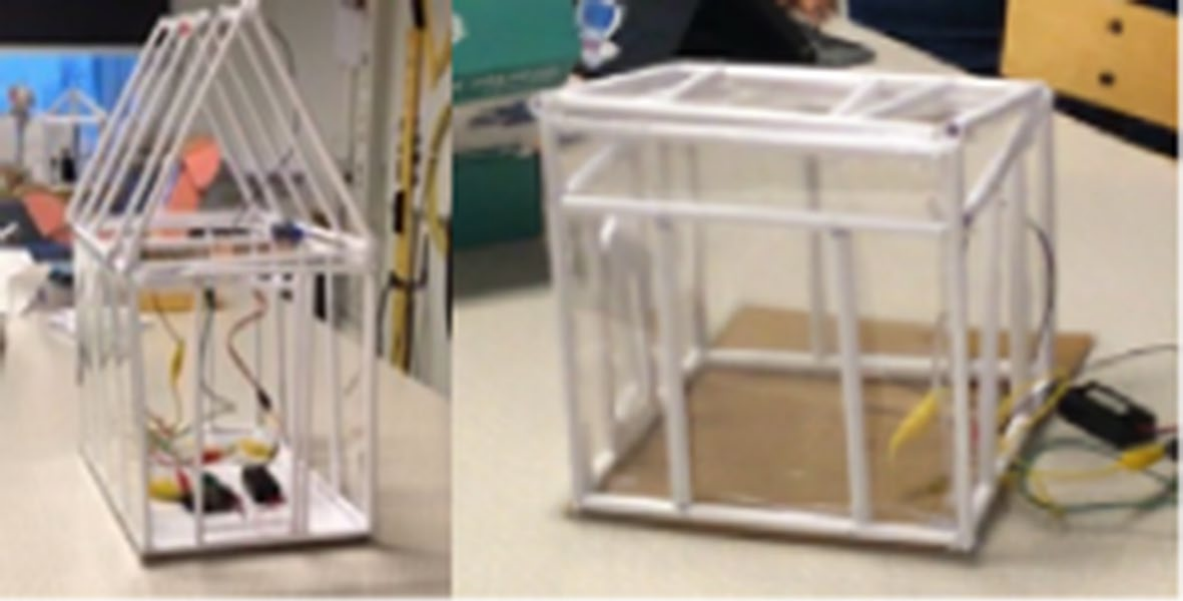
Miniature greenhouses

When constructing the frame of the greenhouse, they were instructed to use rolled office paper, see Fig. 3. They used transparent plastic paper as glass walls. As well as everyday materials, the students also had access to a wide range of electric engines, servo motors, LEDs and other micro:bit components for constructing different functions. The groups had access to micro:bits and all other materials throughout the whole project. The tools available to the students included everyday tools like scissors, glue guns, pliers and knives. All students had their own iPad, which could be used for programming the micro:bits. However, drawing programs were not available on the students’ iPads. Throughout the project, each lesson started with a short reminder of the aim of the project. The teacher established that all groups should have one student who was responsible for the programming, one for the construction, one for designing and one for writing. The teacher urged them to work together and to help each other in the groups. All students had a digital logbook in which they should reflect on their own work process and that of the group.

The teacher was an experienced technology teacher. However, the project was new to both the teacher and the students, but the teacher had recently completed a course in programming for in-service teachers. She had also tried building a miniature greenhouse of her own at home and was prepared for the project.

### The bridge case

The third case is a 7th grade class (students aged 13) involved in designing bridges. The project lasted for four weeks, with two 50-min lessons per week. The project involved designing a miniature wooden bridge which was supposed to be a suspension bridge between two school desks. This assignment was well defined in terms of the specifications to be met. During the introduction, the teacher specified that the width between the desks would be 24 cm and that the bridge must be able to support a weight of 700 g for at least ten seconds. The project was organized as a competition, where the lightest bridge which could support the weight would be the winner. The project ended with a lesson where the students explained and justified their choices of materials and structure. The teacher then tested the bridges. After the test, the students evaluated the different bridges, especially the winning one, from a structural and constructional point of view.

**Fig. 4:**
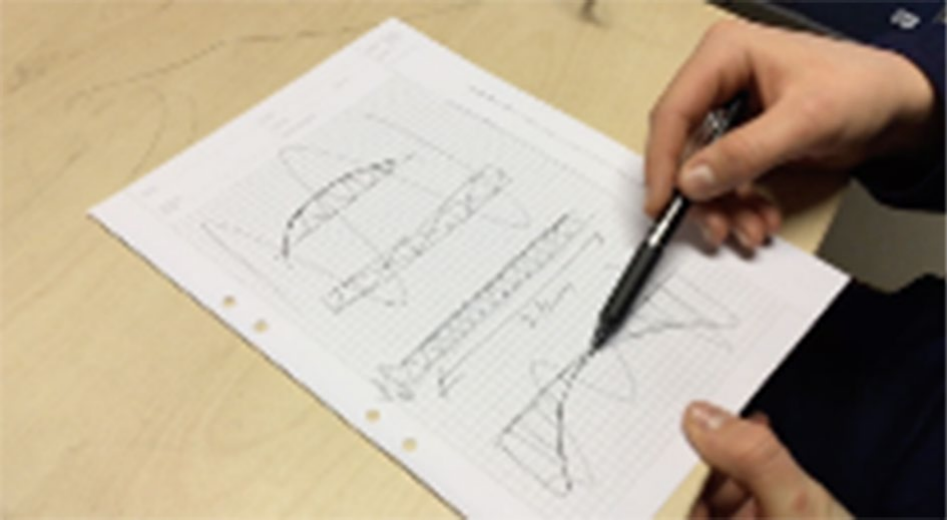
A student making sketches of a bridge

This type of bridge project is quite common in Swedish schools and is usually performed during grade 7. However, this project had a twist: the materials used were converted into points and non-sustainable materials, like plastics, were more expensive to use. The cost and the weight of the bridge were converted into points, and the bridge with the fewest points won. The students worked in groups of three. The materials used for building the bridge included everyday materials like wooden sticks, ice-cream sticks, glue, cardboard, textile cord, plastic and small metal pieces. The students had access to everyday tools like scissors, pliers, saws and glue guns. The teacher observed in this case was fully licensed and experienced, and had worked with the project many times before. Before this project, the students had worked with structures, materials and how to build a stable construction which were used when sketching solutions to the bridge (Fig. 4).

### Ethical considerations

This study follows the ethical guideline of the Swedish Research Council (Vetenskapsrådet, 2017). The teachers, students and guardians were given information about the project. Written consent was collected from the students’ guardians. Students who did not want to participate in the project were positioned beyond the video-recording angle.

## The analysis

As stated earlier, this study aims to answer two research questions: Which activities are explicated in relation to modelling in design activities? What role do modelling activities play in a design project?

To answer the questions, a conventional content analysis is used (Graneheim & Lundman, 2004; Hsieh & Shannon, 2005). A conventional content analysis is generally used when the aim is to describe a phenomenon – here, the activities using models in a design project. It is an analysis in which the researchers immerse themselves in the data and let the categories emerge from the data, described as an inductive category development (see also Liu, 2016).

The video recordings from the three Swedish technology classrooms were analysed using an iterative process, revisiting the data many times. Given the fact that the researchers were not able to attend the lessons in the Greenhouse case or the Bridge case, the first step was to become acquainted with the different classrooms and the activities. Thus, the analysis started by watching the recordings several times to learn more about the context.

In order to answer our research questions, we conducted an inductive analysis of the video recordings, searching for sequences where models were being discussed, both explicitly but also – most commonly – implicitly. The sequences described how models were used in education when working with the design project. In each sequence there was a connection between the model and a description or a term indicating an activity that should be performed. For example, the teacher instructed the students to draw a sketch of the artefact to document their process. In this case, the model is a sketch and the activity is documenting.

The next step was to transcribe excerpts from the selected video sequences and categorize them into different contexts where they were used, for example communicating, planning, constructing, etc. To ensure trustworthiness, this step was conducted by the first author of this paper. Then, the categories were discussed among all three authors and in a discussion between all authors the different categories were divided into subcategories. The category of planning, for example, was divided into the sub-categories identifying problems and transforming. A table with categories, subcategories, excerpts and a description of how models were used in the different categories was produced. The table made it possible to identify activities in which models were used, for example *identifying problems*, *researching the problem* and *planning for constructing*.

The discussions of activities sometimes involve only the teacher, giving directions or instructions to the students, and sometimes discussions between the teacher and students. The analysis was performed in collaboration by all three authors of this paper. The selection of excerpts from the video recordings resulted in about 120 excerpts connected to using models in an activity. The discussions mostly concerned how to use models when carrying out an activity. The transcribed excerpts were translated from Swedish into English, keeping the sense of the utterances rather than producing a word-by-word translation. The analysis resulted in seven different activities involving using models. Not all of the seven activities were present in every context, and some activities were present more frequently during the introduction than during the final lesson, and vice versa.

### Limitations of the study

Since this study is based on three quite different design projects, in three different grades, with different numbers of recordings (Table 1), it is not possible to make direct comparisons between the three cases. Rather, they represent a variety of how models are used in a design project. The differences are interesting when examining the activities which are implemented in the projects. Therefore, the results contribute insights with respect to the type of project and which activities are being discussed. However, since the results are based on three different cases, they also provide an opportunity to ensure the trustworthiness of the findings.

**Table 1:** The three recorded cases

Case	Recorded	Total lesson for project	Total minutes recorded	Equipment for recording	Responsible for recording
Pedometer	2	8X60 min	110	iPad and robot	Researcher
Greenhouse	3	10X60 min	145	iPad and robot	Teacher 2
Bridge	8 within 3 lessons	8X50 min	43*	iPad	Teacher 3

*The teacher chose to record segment of lessons instead of whole lesson

Another limitation is that the Pedometer case only consisted of two recordings, and the last lesson when the students were due to display their solutions could not be recorded. This means that some excerpts regarding the evaluation of the models are potentially missing from the study. However, the material from lessons one and two in the Pedometer case is very rich in data, and lesson three might have been difficult to record anyway, since the students displayed their models in the school library, many people visiting, which could have led to ethical concerns with respect to video recording.

The third potential limitation is that six out of the eight recordings were captured by the teachers themselves. Setting up the equipment sometimes took up to a couple of minutes of the lesson, and on a few occasions the camera did not follow the teacher correctly. If one of the researchers could have been present, he or she could have adjusted this quickly. This may possibly have led to some data loss. However, as we found that the data are rich anyway, we believe that this would not have changed the overall results of our study. Furthermore, knowledge about the context of the education could have been even better with a researcher in place when recording. On the other hand, the absence of a researcher in the room could have made the teacher and students more comfortable and relaxed, resulting in more natural data. In any case, the Covid-19 situation forced us to find a creative way to continue the project.

## Results

By analysing the recordings for excerpts connected to the use of models, we were able to identify seven different activities: *identifying problems*, *planning for modelling*, *documenting*, *constructing*, *testing*, *demonstrating* and *evaluating* (Table 2). Some activities also have sub-activities, as described below, in relation to each activity. In the following sections we describe the seven activities and their sub-activities in chronological order as they appeared in the design process, and exemplify them with excerpts. We also describe the use of the different activities in the three cases by highlighting differences and similarities between the cases. When analysing the recordings, three dimensions within the models were found in all three cases and in all activities: *structure*, *function* and *materials*. The structure is connected to the building technique used when the students construct the model. The function is related to both the function of the whole model or the sub-functions of the model, and the intended functions of the final product. Materials are related to the physical materials used when constructing the model as well as the intended materials of the final product. The three different dimensions are prominent depending on the aims, presumptions and conditions of the project in which models are used. All three dimensions are connected to *knowledge* by the seven different activities.

**Table 2:** Number of excerpts connected to activities involving models

Activity involving models	Number of excerpts
Identifying problems	11
Planning for modelling	24
Documenting	14
Contructing	37
Testing	10
Demonstrating	10
Evaluating	15

### Identifying problems

This activity involves finding and understanding the different presumptions, specifications and aims of the final product. The students have to think as both the *designer* who is constructing the model and the *user* of the final product.

Identifying problems is a common activity during lesson one, especially in the Pedometer case where there were multiple specifications and presumptions to consider when designing the pedometer. When introducing the project to the class, the teacher talks about the design process as the “technology development cycle” (Teknikföretagen, 2021). The cycle contains one step called “needs”, and when explaining this step to the class the teacher emphasizes the specifications that the students MUST consider when designing.

*Teacher one* We have some design criteria as well. It (the pedometer) should suit young people, it should motivate them to walk 10,000 steps a day, it should be easy to carry or wear if they want to. It should be user-friendly and aesthetically pleasing. Finally, you need to think about sustainability. (The Pedometer case, lesson one)

In this excerpt, the teacher talks about the specifications of the final product (referred to as “it” in the above excerpt), of which the students will create a representation in the form of a 3D model. It is important to remember that the final product will not appear physically; it is something that the students develop in their minds as a cognitive model and relate to when thinking about their physical model. When identifying problems, the students have to consider – from both designer and user views – the structure (easy to wear and aesthetics), functions (user friendly, motivational) and materials (sustainability) of the model. The complexity of the project, with many different criteria, makes identifying the problem prominent in lesson one of the Pedometer case.

The Greenhouse case had a rather straightforward aim, namely to build a model of a miniature greenhouse with functions controlled by a micro:bit. The project had few specifications, and when constructing the greenhouse all groups were supposed to use predetermined materials for the frame and “glass” of the house. The project had no clear user, and the only presumptions were that the size of the bottom plate should fit on an A4 sheet of paper. In this way, the material and structure of the model were not negotiable. However, the functions in the miniature greenhouse controlled by the micro:bit were open to students’ own choice and were thus part of identifying problems.

In the Bridge case, the specifications and presumptions were quite clear. The students were only to consider the structure of the bridge in order to make it as stable as possible. The material and functions were predetermined.

The activity of identifying problems varies between the three cases, depending on the extent to which the structure, function and material are predetermined. Whether the user of the final product is known or unknown is also important in relation to identifying problems. With an unknown user, the students only take the designer’s perspective. If the user is known, they also have to take the user’s perspective. Students have to think about the model they are going to build and its structure, functions and materials, and about the final product, which will not appear physically, and the specifications for this. We interpret having both a model and a final product in mind when designing as a challenge for the students.

### Planning for modelling

The *planning for the modelling* activity was used in all three cases, and includes the sub-activities *researching the problem* and *organizing the modelling*. The three main aspects to consider when planning for constructing are structure, function and material. The three cases are different in character, which affects the degree to which material, function and structure are focused on in the planning activity.

#### Planning for modelling: researching the problem

The sub-activity *researching the problem* includes searching for inspiration for cognitive models as well as for constructing the 3D models. Existing similar products are explored, examined and may be implemented into the students’ models. The students searched the internet for products, such as greenhouses, pedometers or bridges, in order to be inspired and to find special technical solutions that could be applied in their models. In the Pedometer case, students needed to investigate all three dimensions: structure, functions and materials. In the two other cases, one or two dimensions need to be investigated due to the design of these projects.

During lesson two in the Bridge case, a group of students are planning the construction of their bridge by sketching and searching the internet. The aim of researching the problem is to find the best structure for the bridge. The teacher is asking the students about a technical solution in their sketch, and one student is using the sketch to explain why the solution is not good.

*Teacher three* How did you get your inspiration for this solution?

*Student one* We searched for bridges on the internet and looked at pictures.

(The Bridge case, lesson two)

The group of students is using pictures of real-life bridges in order to plan the construction, i.e. the structure of their bridge. The pictures, which they found on the internet, are considered models of different solutions, which – combined with their own conceptual models – are transformed into sketches and drawings by the students. As in the Greenhouse case, the students research the problem in order to solve problems relating to controlling functions in the greenhouse.

When searching the internet for inspiration on how to construct different functions, the students often find a program for controlling the function on the same site. The main focus of researching the problem in the Greenhouse case is to find solutions to functions as well as the structure.

#### Planning for modelling: organizing the modelling

What to do and in which order the process is performed is a way of organizing the modelling. In all three cases, the activity where students think through the work process is highlighted as important by the teachers. The teachers often refer to “thinking” within the activity planning. When talking about planning and time for performing the project, teacher one emphasizes different parts of the work process.

*Teacher one* So you need to think, you need to build and you need to program. (The Pedometer case, lesson one)

The students have to plan for building their model and at the same time plan the final product. Our interpretation is that, when talking about the need “to think”, the teacher is referring to a part of the design process that includes planning the construction of the model before starting to build, referred to as *organizing the modelling*. Activities like taking inspiration from other models or products, working on a conceptual model, sketching, planning the work process of construction and choosing materials are included in “to think”. When the teacher expresses the concept “to build”, we interpret this as referring to the actual building process of the 3D model, thus when using materials and tools in order to create a model. The programming part refers to controlling different functions in the model using a micro:bit, as described under the activity *constructing*.

The degree to which the students have to consider the dimensions of material, function and structure when planning for construction is closely connected to how the project is formulated and to what degree the teacher has predetermined factors that influence the three dimensions. In the Pedometer case, students need to investigate and plan in relation to all three dimensions. In the Greenhouse case, the students need to consider the function and to some extent the structure when planning the construction. In the Bridge case, it is only the structural dimension that needs to be considered when planning for modelling. The teacher can decide the degree of difficulty of the task and thus control how much time the activity may take by limiting, predetermining or not displaying material, functions or structure.

### Documenting

The three teachers emphasize that documenting is important in order to describe different steps in the modelling and the model as a representation of the final product. This includes documenting problems encountered during construction, describing the intentions of the final product and documenting during evaluation. The teachers encourage the students to document their work progress and work process in the project with the intention of documenting the development of the models in the form of sketches, drawings and 3D models regarding structures, functions and materials. In all three cases, documentation was carried out in digital logbooks. The students were able to add pictures of sketches, drawings and the 3D model to the logbooks. The teachers had access to the students’ logbooks in order to read them for evaluation and assessment.

#### Documenting: describing problems and solutions

When documenting, there was a particular focus on problems encountered when constructing the model and how they were solved. During the introduction in the first lesson in the Pedometer case, the teacher describes the documentation by referring to another school subject where the students often use logbooks.

*Teacher one* Just as we do in craft lessons, I want you to document your work. What problems have you encountered? How did you solve them? We will end each lesson by writing in the logbook.

(The Pedometer case, lesson one)

The teacher emphasizes the importance of documenting problems, and the way they are solved, when designing the model. Our interpretation is that the teacher wants the students to notice the design process and its various steps, especially problem-solving parts where students try, evaluate and retry different solutions using the model. These sub-activities, used mostly in the Pedometer case and the Greenhouse project, focus on documenting the influence of the function and structure of the model during the design process.

#### Documenting: describing intentions of the final product

As described above, certain attributes and functions of the final product were difficult to represent in the students’ models because the materials were too simple and the students lacked resources and skills. This emphasizes the activity of documenting in order to describe the students’ intended intentions with the final product. Teacher one expresses the need to describe certain functions in a conversation with a student during practical work in the classroom.

*Teacher one* You can of course make the pedometer send messages to an app as well… But we do not develop the app, but you describe how you think it will work instead. You describe what the app should do instead of getting stuck in the programming. (The Pedometer case, lesson two)

There is a focus on the 3D model of the pedometer and its functions when discussing how the documentation can strengthen the description of the intended final product. The students describe the intended appearance and functions of the final product in words, and sometimes with visual models. This activity is common only in the Pedometer case, due to the complexity of the project and the lack of different materials for building. The focus is mostly on materials and functions where differences occur between the final product and the model built by the students.

#### Documenting: evaluating the model

Documenting is also used as an activity in the evaluation of the model. The students in the Bridge case use the 3D model of the winning bridge to document their thoughts about why the structure of a technical solution is advantageous. In the last lesson of the Bridge case, the teacher encourages the students to document their analysis of the winning bridge. The teacher shows the class the physical model of the winning bridge.

*Teacher three* Then we enter the analysis phase, you use Classroom [a digital platform] and create your own document. This is where you write the analysis. Why was this the winning bridge? What was it about this bridge that made it easy to build but still strong? Look at the bridge and analyse it. (The Bride Case, lesson three)

The 3D model of the winning bridge is used to get the students to document their own analysis of the structure and its stability. We interpret this as meaning that the teacher wants the students to practise analysing and documenting using a 3D model, and to compare the structure of the winning bridge model with their own solution, thereby challenging their understanding of the structure of models.

The activity of documenting is used in various ways depending on the case and its presumptions, constrictions and ways of presenting the solution. The activity of documenting is present in all three cases when describing problems and solutions during the construction of the model, but when the complexity of the structure and function of the final product is high and the building materials are simple, the students use documenting to explain the final product’s intentions regarding materials, functions and structure. These intentions are difficult to show in the model when using simple materials, and documentation is used as a complement when presenting and marketing the final product using the model. Once more, the students have to think about the final product’s functions, structure and materials, and about what can be expressed in the model.

### Constructing

The constructing activity concerns building a 3D model using special techniques, often in the form of a mock-up, and designing programs for controlling certain functions in a 3D model.

When constructing a physical model, the building material used is of great importance and affects both the structure and the function of the 3D model. The schools normally only have access to simpler, cheaper everyday materials, which may affect building techniques as well as the authenticity and accuracy of the model. When constructing the greenhouse, the students use everyday materials and a special construction technique to make the frame strong. During a joint review, the teacher uses office paper and wooden sticks to teach the students how to roll the paper using the sticks to get a strong frame.

*Teacher two* Your building material is office paper. I have tested rolling the paper diagonally, and it’s the easiest way. The most important thing is that you actually try to tighten it up when rolling the paper. Then you get a bit like a cardboard straw and they are actually very strong. (The Greenhouse case, lesson one)

The materials are predetermined in the Greenhouse case, which means that the students do not have to consider which materials are most suitable. In the Bridge case, the prerequisites are similar: the materials are predetermined as wooden sticks. In the pedometer case, the students can choose from materials they come across at home or the existing materials at school. In cases where the material is predetermined, students are given the opportunity to focus on developing an understanding of the model’s structure.

The 3D model could be fully functional, functional in certain aspects or a mock-up of the intended product. Materials, structure and functions are the aspects being manipulated when constructing the model. The use of everyday materials can be limiting when constructing the model, often leading to the model being a mock-up of the intended product. For instance, instead of making a pedometer with a rubber wrist strap, the students use cardboard.

*Teacher one* A rubber wrist strap? I don’t have that… but you are building a model. I think you can make a model in cardboard and then you can write (in your logbook) that it is a different material. (The Pedometer case, lesson two)

The teacher refers to the fact that the student is building a model and that it is not constructed using exactly the same materials as the final product. She suggests that the student should build the model in cardboard and describe the intended appearance in the documentation, in order for the intentions to be understood by people evaluating the solution. We interpret that because of the problems when building models, the teacher encourages the students to document all the intentions and functions of the final product but build only some of them.

Building a mock-up is common, especially in the Pedometer case, where it serves as a proof of concept to show the students’ ideas and intentions when they present their model at the end of the project. The pedometer is controlled by a micro:bit which affects the structure of the model, making it rather large (Fig. 2) compared to the intended product. This results in students often building a scale model. The micro:bit has limitations, making the model simpler and often scaled compared to the intended product. The teacher frequently refers to how they are constructing a model and that the final product will have a different structure. When discussing the scale of the model with a student, the teacher uses a micro:bit to explain.

Teacher one: And this (shows micro:bit) you can think of as just part of the display. You could make it smaller when it goes into production… So you do it as a model of what it will look like. Then you can of course produce it on a smaller scale. (The Pedometer case, lesson two)

The teacher uses the micro:bit to discuss the scale of the model. She mentions that the model shows what the final product will “look like”. Our interpretation is that the teacher is referring to the form of the product but not at the actual scale. Functions are sometimes too difficult for the students to construct, which means that they have to use a mock-up in their model.

### Testing

This activity is used to find the optimal fit for purpose regarding the materials, structures and functions of the model. In the Pedometer case and the Greenhouse project, the models are supposed to have one or more functions controlled by programming a micro:bit. In order to get the functions to work properly, the students have to test them several times. In the Greenhouse case, some students have coded a program for the temperature sensor that they intend to have in their greenhouse. The students are eager to test their program, but the teacher advises them to test the program by using a light sensor instead of a temperature sensor because it is much easier.

*Teacher two* To test if your program is working, you can do this: Choose light level instead of temperature and test the function. You can change it back to temperature later on. (The Greenhouse case, lesson two)

When testing various functions in the greenhouse, some are more complicated to test than others. The teacher suggests that the students use an easier function to test their program. We interpret that she does this in order to save time for the students, but also to show that different functions can be used to test whether the code is correct in the program. The model, and the functions displayed in the model, develop the students’ knowledge by testing different solutions. We interpret the purpose of this as a process where the model is being used to reach a final product that meets expectations and presumptions in the final product.

In the Bridge case, there is extensive testing which can be connected to the fact that the materials and functions are fixed and that the structure built by the students is the object of testing. The students had some knowledge about durable and stable structures, and commonly used that knowledge in combination with inspiration from other bridges in the testing activity. The students build, test and rebuild the model in order to find the best solution for structure, taking the weight of the construction into account.

*Teacher three* What are you thinking right now?

*Student 4* That we will change the bridge a bit. We thought about putting on weight like this, but it didn’t work… (student demonstrates in 3D model)

Teacher three: Can’t you have it the other way around?

*Student 4* No, because then there will be problems here (student turns around the model to demonstrate)

(The Bridge case, lesson two)

The students and the teacher use the 3D model to discuss the testing of the bridge. Our interpretation is that the students have tested the model with a weight on it and observed weak points in the construction. Since the students have tested the bridge, the discussion with the teacher is more detailed and they are able to show the exact problem areas in the construction. Because the material is restricted to wood, the testing is of the structure rather than the material. Our interpretation is that the teacher uses the model to recall prior theoretical knowledge about structures and stable constructions, and to put this into practice.

### Demonstrating

The activity of demonstrating is about using a model to present or market a product. In the Greenhouse case, the students are supposed to demonstrate their greenhouse with its different functions and structures using their model. When starting a lesson, the teacher explains to the class how to demonstrate their greenhouse using the model.

*Teacher two* When presenting your greenhouse, you should show what the greenhouse looks like, explain why you chose to build it as you did and how you control the different functions in it. (The Greenhouse case, lesson two)

We interpret that the teacher is referring to the structure of the greenhouse when she talks about what the greenhouse looks like and why the students chose to build it as they did. When talking about how the different functions are controlled, she refers to the functions in the greenhouse. The materials are mostly predetermined in the Greenhouse case. In the Pedometer case, the fictional visit from the Swedish National Board of Health and Welfare at the end of the project puts more weight on the students’ presentations of their products. An exhibition with all the 3D models of the pedometers was supposed to be visited by a group of teachers acting in the role of the external visitors. When introducing the project, teacher one explains about the exhibition at the end of the project.

*Teacher one* Then you have to promote it, the National Board of Health and Welfare comes here, and you must market your product. And then we’ll see which one they choose.

(The Pedometer case, lesson one)

Our interpretation is that the teacher is referring to the model of the pedometer when using “it”, and that the exhibition is a way of making the project more authentic. Furthermore, the teacher uses the term “market”, which we perceive as more extensive than simply a presentation to the class. Since the Pedometer case is rather open, the students are required to demonstrate structure, functions and materials using their model. The demonstration is made more difficult because the 3D model is a mock-up of the final product in terms of its structure, functions and materials. The 3D model must represent the intended product as closely as possible despite the use of everyday materials, which also demands an extensive demonstration from the students during the exhibition.

### Evaluating

This activity is about how to use the students’ finished 3D models at the end of the project in order to evaluate the technological solutions’ fit for purpose. The 3D model is often the object of this activity, but a visual model – like a sketch or a drawing – is sometimes used. Evaluation of materials is not used due to predetermined materials in the Greenhouse and Bridge cases and a small range of materials to choose from in the Pedometer case.

When evaluating a model in the Bridge case, we interpret that the students use their theoretical knowledge to evaluate the bridge and to develop their own cognitive models of technical solutions. In the Pedometer case and the Greenhouse case, the development of the students’ cognitive models is not obvious. In the Bridge case, the importance of analysis is expressed by the teacher during the introduction to the project.

*Teacher three* Then you should do an analysis of the winning bridge. What was it in the construction that was successful and how could it be built so lightweight? This analysis is important. You use parts that you have learned to analyse in theory. (The Bridge case, lesson one)

In the Bridge case, almost a whole lesson was devoted to evaluation. The focus was on the structure during this evaluation, since both function and materials were more or less predetermined. The teacher had a discussion in the class, in which they evaluated the different structural solutions in the bridges, especially the winning bridge. Since the project was like a competition where the lightest bridge that could carry the predetermined weight won, the discussion about the structure was at quite an advanced level.

*Student 5* Maybe it’s how big the angle is on the stays. (student demonstrates an angle with his hands)

Teacher three: Exactly, it makes a difference whether you have it this way or that way. What do you think will be the difference between them? (draws a 120 degree and a 30 degree angle on the board)

*Student 6* Strength?

*Teacher three* That’s right, so if we increase the height as in this angle (demonstrates 30 degree angle), the strength also increases based on the load coming from above. (The Bridge case, lesson three)

The teacher uses the 3D model and also a visual model (drawn on the blackboard) of the winning bridge to show the students how increasing the height of a specific part of the bridge can enhance the strength. We interpret that by using both the 3D model and a simplified visual model of the bridge, the students are better able to see the importance of the structure for the strength and stability of the model. The evaluation in the Greenhouse case was focused on function rather than structure. We interpret that most of the greenhouse structure was similar to a full-scale greenhouse, which led to less variation in structure and less discussion during evaluation. This can be seen in the excerpt below.

In the Greenhouse case, an evaluation of the greenhouses was performed through a discussion between the students and teacher when the 3D model of the greenhouse was demonstrated in front of the class. The different functions controlled by the micro:bit were evaluated. The teacher also emphasizes that the students should consider the intentions of different solutions and functions when evaluating the model.

*Teacher two* You have built your greenhouse in a classic form. What kind of digitization do you have in your greenhouse?

*Student 7* We have a thermometer connected to this door. (student points to 3D model)

*Teacher two* Is it activated by pressing it?

*Student 7* Yes, that’s right. (student demonstrates the function)

*Teacher two* There are some adjustments to be made, but what I want to know is: What was the thought behind it? (The Greenhouse case, lesson three)

We interpret that the evaluation of the greenhouse is focused on the different functions being controlled by the micro:bit, and that when the teacher asks about different solutions and the students demonstrate them, this is part of the evaluation. The teacher is also evaluating the functions when she says “There are some adjustments to be made”. Our interpretation is that the function is not working perfectly, which leads to the teacher’s comment about adjustments. Considering that the evaluation takes place in front of the class, the teacher has to consider how she expresses herself about the model being displayed so that the students will not lose face in front of their peers.

## Summary of results

To summarize the main findings of our results, structure, function and materials appear in all activities (Table 3). However, the degree of openness in the design project affects which dimension will appear in the classroom discussions. If the project is open, as in the Pedometer case, where the students had to choose both the structure and the function of their project, all three dimensions were frequently discussed. However, in the Greenhouse case and the Bridge case, the materials and structures were predetermined and it was mostly the function of the models that was elaborated upon. Moreover, if the project was open, the students put a lot of effort into trying to identify the problem and planning for modelling.

**Table 3:** Dimensions of models in activities

Activities	Structure	Function	Materials
Identifying problems	User and designer perspectives on the final product, like size, aesthetics and ergonomics	Functions to satisfy the user controlled by the programmed micro:bit like producing a sound, light or temperature regulation. Thus user and designer perspectives	User and designer perspectives on the final product’s material properties like weight, strength and sustainability
Planning for modelling *Planning for modelling: Researching the problem*	Investigating the structures of existing products (photos and real-life products) and models (visual and 3D models)	Investigating the functions of existing products (photos, videos and real-life products). Coding for programmed functions	Investigating existing (real-life) products’ materials for inspiration
*Planning for modelling: Organizing the modelling*	How to put together the different parts of the model, and in which order		
Documenting *Documenting: Describing problems and solutions*	Problems and the way they are solved, when designing the model		
*Documenting: Describing the intentions of the final product*	Intended appearance (size, shape and colour) of the final product	Intended functions (sound, light and temperature regulation) of the final product	Intended materials in the final product
*Documenting: Evaluating the model*	Evaluate the structure of a model by describing it	Evaluate the functions of a model by describing them	
Constructing	Mock-up of the final product’s structure, such as size, shape or colour	Mock-up of the final product’s functions, such as sound, light or temperature regulation	Mock-up of the final product’s materials
Testing	Testing the model structures for strength, aesthetics, ergonomics and weight	Testing the model’s functions, such as sound, light, temperature regulation or counting steps	
Demonstrating	Marketing or describing the final product’s size, shape, aesthetics or ergonomics	Marketing or describing the final product’s functions such as sound, light, temperature regulation or counting steps	Marketing or describing the final product’s materials
Evaluating	Use theoretical know-ledge to evaluate different structural solutions	Functions in the 3D model are evaluated through testing and discussion	

## Discussion

This study aims to examine explicated models using activities and what role these activities play in a design project. When examining the modelling activities, we find that they are connected to three model dimensions: *structure*, *function* and *material*. In the discussion, we will emphasize three interesting findings from our study and relate them to the dual nature of models (Nia & de Vries, 2017).

### The functions of models in a design project

One finding from this study is that the goal of the design project differs between our three cases. This could be to construct a 3D model as a mock-up of a final product, or to produce a miniature model of a real full-size product in different materials or with different functions. A project with more predetermined factors regarding the model’s dimensions, like the Bridge project, produces a more realistic model that resembles an authentic model. By comparison, a more open project – like in the Pedometer case – leads to the model being more of a mock-up of the final product, where both materials and functions are documented as intended, rather than built into the actual model.

The application of the 3D model differs between the three cases in our study. In the Pedometer case, the function of the 3D model is used to market the final product in an exhibition. This emphasizes the importance of documenting the intended structure, functions and materials of the final product in order to communicate, demonstrate and make a decision about the final product. This result is in line with the findings of Yrjönsuuri et al. (2019), which show that prototypes serve a role in students’ verbalization and demonstration of ideas in the design process. In the Greenhouse case, the 3D model is the final product since the project is about designing a miniature greenhouse with sub-functions controlled by a micro:bit. However the model is a mock-up in certain respects since not every intended function in the greenhouse is actually built by the students. For example, some functions controlled by the micro:bit were too hard for the students to program. Regarding the Bridge case, the 3D model is also the final product since the aim was to build a model bridge.

The differences between the three cases, their presumptions and the application of the 3D model indicate a connection between the character of the project and the function of the 3D model. An open project, like the Pedometer case, leads to more considerations for students when constructing the model. They have to consider both the final product’s structure, functions and materials and which structure, function and materials to show in the 3D model used to demonstrate and market the final product. The process of constructing the model is difficult and time-consuming. In a project with one or two predetermined model dimensions, like the Bridge case, the students only have to consider structure, which is less time-consuming and allows them to concentrate on one dimension. The ways in which the model is actually used in the three cases are different. In the Pedometer case, the model is used to enhance both the functions and structure of the final product. Together with the documentation describing the intentions of the final product, they give the decision-makers a chance to evaluate the final prototype. In the Bridge case, the model is used to enhance the structure in order to understand and learn more about the structure and stability of constructions. In the Greenhouse project, the model of the greenhouse allows the students to learn about controlling functions by programming a microcontroller. The model is constructed mostly for the purpose of accommodating the sub-functions controlled by the micro:bit.

The results from this study, performed in a classroom setting, show that models play a crucial role in the three design projects. This can be contrasted to the teachers’ own descriptions of how they use models (Citrohn & Svensson, 2020). In the interviews, the teachers do not use models for problem-solving or evaluation. However, our results show that in an actual teaching situation, activities such as evaluating and describing problems and solutions are present in the classroom dialogue. This indicates that more is happening in the classroom than the teachers themselves reflect on in an interview situation.

### The character of a design project

A school design project can have different characteristics, depending on presumptions regarding for example material, time, teachers’ and students’ knowledge, information about users and the aim of the project. The characteristics are also affected by teachers' understanding of how to use the design process as a model for solving technological problems. The two extremes are a fully open undetermined project and a fully predetermined closed project. An open project where structure, function and materials are undetermined is more demanding for students than a closed project where functions, materials and structure are predetermined.

The Pedometer case is fundamentally an open problem, creating a pedometer for young people that inspires them to walk. The only restriction is that a micro:bit should count the steps. In theory, this could lead to a great variety of models with many different materials, structures and functions. However, as mentioned before, *materials* are rather restricted, which – together with the size of the micro:bit – affects the *structure* of the model of the pedometer. Most students used cardboard, which restricts the structure to being quite square in shape, thus a Soft Model (Isa & Liem, 2014). The micro:bit controller is also a limitation with respect to which functions can be coded and which functions the students had sufficient knowledge to code. This leads to students building a mock-up of their intended product. The Pedometer case is demanding for students, since they have to think about both the model and the final product. Svenningsson (2019) states that if the teacher provides the students with a variety of materials, they will be encouraged to test different solutions to their design problem. In line with this, the Pedometer case enables the students to use their creativity. Even though they are restricted by the rather limited supply of different materials, they were allowed to move outside these restrictions by building a mock-up and adding their descriptions of other materials that they wanted to use in their final product.

The final product’s connection to the model is also interesting. In a project like the Bridge case, with few open dimensions of models, the model is actually the final product which means that no mock-up of the final product is needed. Once more, the greenhouse is somewhere in between the Pedometer case and the Bridge case. The size of the greenhouse is the final product, while some functions and materials are mock-ups. In the Pedometer case, most of the model is a mock-up of the real product.

We conclude that it is important for a teacher to be aware that the degree of openness of the design project will affect the potential learning outcome. Both Svenningsson (2019) and Yrjönsuuri et al. (2019) highlight the importance of the teacher’s project planning. They do so mainly from a material angle. The materials at hand will affect the activities taking place in the technology education classroom. However, we would also add the importance of taking the openness of the project into account when planning these kinds of design projects. On the other hand an open project can be designed with the intention of teaching about the design process. The design process itself can be the product being taught. In this study this is not clear but our focus is on the models and modelling not on the design process. We will now dig deeper into the three different model dimensions: function, structure and materials.

### Knowledge in the dimensions of function, structure and materials

The dimensions of function, structure and materials – see Table 3 for an overview – are about examining, using and evaluating functions in models. Regarding the *dimension of function*, the students are supposed to have knowledge about more functions than the ones included in their model since they are asked to describe the final product, which usually consists of more functions, in documentation or when demonstrating the model. Thus, the students often need knowledge in order to build a mock-up of the final product regarding functions. The students also need knowledge of what the functions in the models or the final products are supposed to *achieve*, like opening a window when the temperature gets too high in the Greenhouse project or rewarding a user with a tune when they walk 10,000 steps. This knowledge is used during modelling activities as well as when describing the final product. Finally, the students need knowledge about which artefacts to use and how to use them, and thus how the artefacts are supposed to be activated, connected and controlled in order to get the functions to work. The *dimension of structure* includes knowledge about stability, sustainability and methods for joining materials, as well as aesthetics, ergonomics and the appearance of models, in order to demonstrate and evaluate the final product using the model. Once again, the student needs knowledge about how to build and construct the model, and about the structure of the final product. The *dimension of function* and the *dimension of structure* are connected to all seven modelling activities – see Table 3 – while the *dimension of materials* is connected to the fewest activities since materials were predetermined in some way in all three projects. In this dimension, the students have to think about the materials of both the model and the final product without having adequate knowledge about materials. We interpret a weak connection between materials and structure/function in the students’ knowledge.

Regarding our results for the three dimensions of models and the activities, our study complements the framework of the dual nature of models by Nia and de Vries (2013). While the dual nature of models is based on a literature review (of both science and technology) and an investigation of curricula, our study is based on video recordings of technology lessons. Thus, our results serve as en empirical expansion to the theory of the dual nature of models. 

**Fig. 5:**
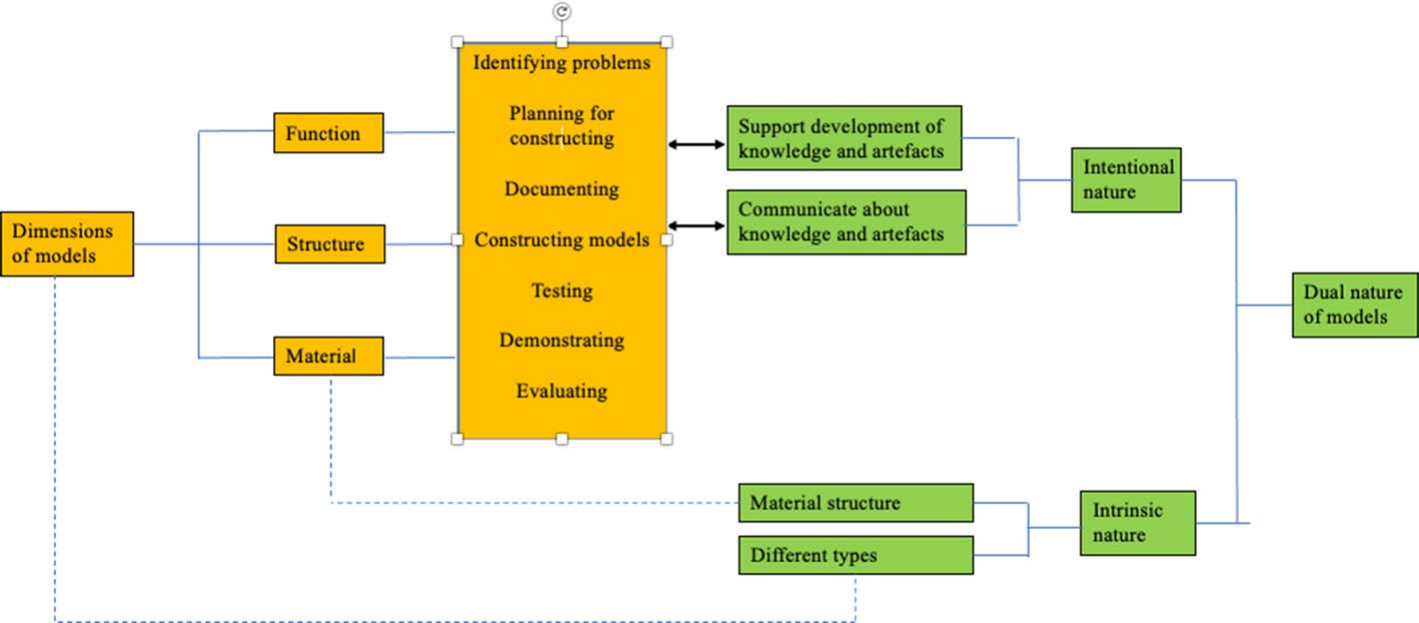
The framework of the dual nature of models and links to the results from our study

The seven modelling activities identified in this study are connected to the categories of *support development of knowledge* and *communicate about knowledge and artefacts* in the dual nature of models (Nia & de Vries, 2017), see Fig. 5. We also identify a connection between the dimension of material and Nia and de Vries’ material structure.

## Conclusions and implications

This study shows that the students are given different possibilities of learning about models and modelling depending on the teacher’s design of the project. We have identified seven different activities where teachers and students talk about models and modelling in relation to the design project. In a project with a lower degree of freedom, the students only have to consider the model as the final product. This applies to both the Greenhouse case and the Bridge case. Neither of these projects have expressed final users, and hence the students only have to think as the designer, leading to a lower level of complexity. Therefore, the degree of freedom in a project will predict the complexity for the student. However, our results also indicate that a lower degree of freedom provides opportunities for deeper discussions on one of the three dimensions and allows the students to generalize their findings from the design project. It is important to consider the teacher’s intentions, when discussing the character of the design project. The teacher might have special intentions of the modelling, like applying theoretical knowledge about strength and materials when constructing or teaching the students to apply the design process when solving a problem. We suggest that this should be interesting to investigate further in a future study.

This study contributes new insights on modelling activities in a classroom setting. We suggest that the three dimensions of models – together with the dual nature of models (Nia & de Vries, 2017) – could be used by teachers as a tool for planning and evaluating design projects regarding complexity and aim. This study emphasizes the importance of clarifying the aim of a design project in technology education. Even though this is only a small-scale study, it contributes important knowledge and insights about teaching in design projects. However, further studies that systematically examine design projects with different characters and their implications on students’ and teachers’ awareness of models and modelling are needed. Also the teachers' intentions of the students' physical models and modelling in a design project needs to be studied in order to know more about the usage of models and modelling in technology education. As mentioned before, the intention of using models in a design project could be to learn more about the design process itself or to use models in order to practice theoretical knowledge about strength and materials.
